# Newly Identified Enterovirus C Genotypes, Identified in the Netherlands through Routine Sequencing of All Enteroviruses Detected in Clinical Materials from 2008 to 2015

**DOI:** 10.1128/JCM.00207-16

**Published:** 2016-08-24

**Authors:** Coretta C. Van Leer-Buter, Randy Poelman, Renze Borger, Hubert G. M. Niesters

**Affiliations:** The University of Groningen, University Medical Center Groningen, Department of Medical Microbiology, Division of Clinical Virology, Groningen, the Netherlands; Memorial Sloan Kettering Cancer Center

## Abstract

Enteroviruses (EVs) are a group of human and animal viruses that are capable of causing a variety of clinical syndromes. Different genotypes classified into species can be distinguished on the basis of sequence divergence in the VP1 capsid-coding region. Apparently new genotypes are discovered regularly, often as incidental findings in studies investigating respiratory syndromes or as part of poliovirus surveillance. Recently, some EVs have become recognized as significant respiratory pathogens, and a number of new genotypes belonging to species C have been identified. The circulation of these newly identified species C EVs, such as EV-C104, EV-C105, EV-C109, and EV-C117, nevertheless appears to be limited. In this report, we show the results of routine genotyping of all enteroviruses detected in our tertiary care hospital between January 2008 and April 2015. We detected 365 EVs belonging to 40 genotypes. Interestingly, several newly identified species C EVs were detected during the study period. Sequencing of the 5′ untranslated region (5′ UTR) of these viruses shows divergence in this region, which is a target region in many detection assays.

## INTRODUCTION

Enteroviruses (EVs) are common pathogens that are associated with various clinical syndromes involving different organ systems. Historically, EVs were classified according to clinical, serological, and culture characteristics, but currently, sequence divergence in the VP1 capsid-coding region is used for allocation of these viruses into different genotypes. Together with the closely related rhinoviruses, EV species belong to the genus Enterovirus in the family Picornaviridae (1). To date, 8 EV species are recognized, A to H, of which species A to D are known to cause disease in humans. Strains that have less than 75% nucleotide and 85% amino acid similarity in the VP1 region are classified as different genotypes (2, 3). Newly identified EV strains are regularly reported (4). Infections caused by EVs include hand-foot-and-mouth disease (HFMD), myocarditis, respiratory infections, meningitis, and acute flaccid paralysis (AFP) (1). Although some clinical conditions are classically associated with one or more EV genotypes, such as AFP, which before mass vaccination used to be most commonly caused by polioviruses, it is apparent that each EV type is capable of causing a variety of clinical syndromes and that a particular clinical syndrome may be caused by a variety of EV types. Correspondingly, EV-A71, which causes HFMD, is also capable of causing neurological infections (5). Additionally, neurological infections as well as HFMD can be caused by a variety of EV genotypes (6, 7). Most EV infections are nevertheless subclinical, and their circulation in the population goes unnoticed. International surveillance of EVs is limited but exists for poliovirus. In addition, many countries have a surveillance system at the national level for cases of AFP and neurological infections caused by EVs other than poliovirus (8, 9). Data about circulating EV genotypes are thereby generated, but these data are not comprehensive. EVs, beyond poliovirus, continue to be important pathogens. Indeed, during the summer of 2014, an unprecedented outbreak of EV-D68 occurred, affecting thousands of people in North America as well as in Europe (10, 11). This outbreak highlights first that EV outbreaks are unlikely to remain confined to a single geographical area and second that EVs may be the cause of severe respiratory illness.

Respiratory samples have also been the source from which several new members of the EV-C species have been first identified. EV-C104 was first found in Switzerland in 2005 in respiratory samples from patients with respiratory illnesses. Since then, EV-C104 was found sporadically in Italy, Japan, and Gambia (12–15). EV-C105 was first identified in the fecal sample of a Congolese man with AFP; however, this virus was subsequently detected in respiratory samples in Italy, Peru, Cyprus, New Zealand (16–19), and most recently in another case of AFP in the United States (20). EV-C109 was first identified in respiratory samples in Nicaragua (21). Since its first description, it has been detected in respiratory samples in Italy and Hungary and in a fecal sample in Congo (22–24). EV-C117 was first found in a Lithuanian child with pneumonia (25). However, in total so far, about 25 reported cases of EV-C104 have been described over a period of 10 years, and for the other “new” EV-C species viruses, this number is even lower. Sequence information is limited.

In the present study, we show the results of the routine sequencing strategy applied in our clinical virology laboratory for all EVs detected between January 2008 and April 2015.

## MATERIALS AND METHODS

### Screening of clinical samples.

Our laboratory is part of a tertiary care university hospital. Clinical materials are received from patients who are admitted as well as from patients who visit outpatient clinics. Because the hospital is the country's largest organ transplantation center, two-thirds of its patients are immunocompromised.

All cerebral spinal fluid (CSF) samples from patients with neurological symptoms, respiratory samples from patients with respiratory symptoms, and fecal samples of patients with diarrhea and gastrointestinal symptoms referred to the clinical virology laboratory were screened for EVs. All materials were screened with our routine screening multiplex reverse transcriptase PCR (RT-PCR) assays, which include a minimum of 5 targets in CSF (herpes simplex viruses 1 and 2, varicella-zoster virus, enterovirus, and parechovirus), 14 viral targets in respiratory samples (including Influenza A and B, rhinovirus, coronaviruses, parainfluenza viruses, adenovirus, bocavirus, respiratory syncytial virus [RSV], and human metapneumovirus [HMPV]), and 8 viral targets for fecal samples (adenovirus, bocavirus, rotavirus, astrovirus, sapovirus, norovirus, enterovirus, and parechovirus). In addition, blood (plasma and serum), vesicular fluid, and biopsy specimen materials are tested for EVs when clinical presentations are compatible with enterovirus infections, such as hepatitis, myocarditis, or HFMD. For the detection of enteroviruses, we used a previously published protocol (26).

Patient characteristics (i.e., date of birth and sex) and clinical data (i.e., underlying condition, clinical presentation, and coinfections) were recorded. Repeated samples from the same patient within a 3-week period yielding the same genotype were excluded. Identical EV genotypes from different materials from the same patient were included in the overview of isolates per genotype, but only one isolate per patient per disease episode was included in calculations for overall prevalence of the different EV genotypes.

### Sequencing and phylogenetic analysis.

For the determination of the genotype, we sequenced the VP1 gene as published previously by Nix et al. (27). For molecular analysis, the partial VP1 regions of all 367 EVs were compared with the homologous sequences of prototype strains available in GenBank as previously described (2).

In order to gain more information on the uncommon EV-C genotypes that were detected during this study, partial sequencing of the 5′ untranslated region (5′ UTR) was performed on these strains using primers DK001 (28) and EV-AII (TTCTGIGTIGAIACYTGWGCICCCAT), amplifying a 430- to 570-bp amplicon (nucleotide position 182 to 712, relative to the Tomkins coxsackievirus [CV] A1 strain). We also performed partial sequencing of the 5′ UTR of the four coxsackievirus A21 strains.

Sequencing was performed with an automated DNA sequencer (ABI 3130XL; Applied Biosystems Instrument, Carlsbad, CA, USA) using the BigDye Terminator v3.1 cycle sequencing kit. Sequence data were analyzed using BioNumerics software 6.6 (Applied Maths, Sint-Martens-Latem, Belgium). The 5′-UTR sequences were aligned with enteroviruses A to D as well as with rhinovirus A to C sequences, which were available in GenBank. Subsequently, phylogenetic analysis was performed with the EV-C strains detected in the respiratory samples included in our study using the neighbor-joining algorithm with the nucleotide/Kimura two-parameter method using BioNumerics software. The reliability of the phylogenetic branching patterns was determined by the bootstrap resampling test with 1,000 pseudoreplicates, which are shown as percentages. Sequence similarity was also examined by the unweighted pair group method using average linkages (UPGMA) using BioNumerics software.

### Statistical analysis.

Statistical analysis was done using chi-square tests.

### Accession number(s).

The partial VP1 sequences and 5′-UTR sequences that were determined in this study have been deposited in the GenBank sequence database under accession numbers KT735342 to KT735354 and KT735355 to KT735367.

## RESULTS

Screening 26,390 samples identified EV RNA in 399 samples from 351 individuals. EVs were detected in a variety of samples, including in 105 respiratory samples of 14,565 (0.7%), in 5 out of 54 skin lesions (9.3%), in 67 of 2,106 CSF samples (3.2%), in 214 of 9,510 fecal samples (2.2%), and in 8 of 155 blood samples (5.2%). Subsequently, 365 isolates could be genotyped (92%) from 328 disease episodes. A disease episode was defined as a single clinical period during which virus, as well as clinical symptoms, was present.

### Results of genotyping.

**FIG 1:**
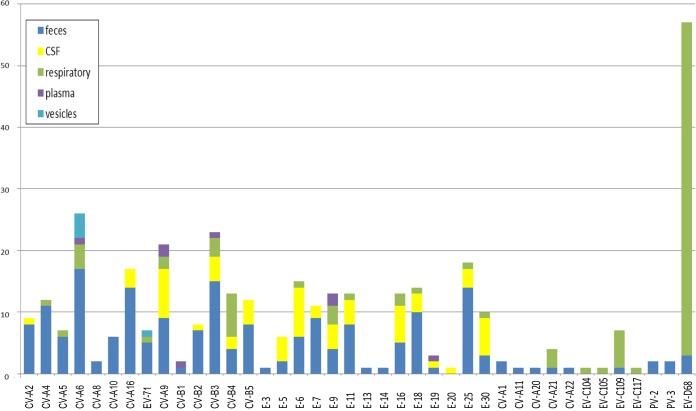
Enterovirus genotypes detected in this study per clinical material from which they were isolated. E, echovirus; EV, enterovirus; CV, coxsackievirus; CSF, cerebrospinal fluid.

Thirty-four detected EVs could not be genotyped. The remaining 365 EV detections were from 65 CSF samples, 5 vesicular fluids, 8 plasma samples, 97 respiratory samples, and 190 fecal samples (Fig. 1).

The EV genotype that was most frequently detected during the study period was EV-D68. The 2 years with EV-D68 upsurges (2010 and 2014) were the years with the most detected EVs overall (Table 1). Even without the number of detected EV-D68, significantly more EVs were detected in 2014 than in the other years (*n* = 61 versus an average of 45; *P* < 0.02).

**TABLE 1:** Number of detected enterovirus genotypes per year, in the Northern part of the Netherlands between January 2008 and April 2015

Genotype	Year								Total no.
	2008	2009	2010	2011	2012	2013	2014	2015	
CV-A2	1			4			4		9
CV-A4	2		2	3		1	4		12
CV-A5	1	1		2		3			7
CV-A6	5			1	3	5	12		26
CV-A8	0	1		1					2
CV-A10	3					2	1		6
CV-A16	5		4	1	4	1	2		17
EV-71	0	1	4			2			7
CV-A9	0		17		2	2			21
CV-B1			1				1		2
CV-B2	1	2			4		1		8
CV-B3	8		1	3		8	3		23
CV-B4		6		1	2		3	1	13
CV-B5	1	6	2		1		2		12
E-3							1		1
E-5				1	5				6
E-6		9			1	1		4	15
E-7	5			6					11
E-9	2	2		5	1	1	2		13
E-11			5		7	1			13
E-13	1								1
E-14			1						1
E-16							13		13
E-18	1		1	4	6		2		14
E-19				3					3
E-20					1				1
E-25	8			6			4		18
E-30			7	1		2			10
CV-A1		1	1						2
CV-A11							1		1
CV-A20						1			1
CV-A21						4			4
CV-A22							1		1
EV-C104							1		1
EV-C105							1		1
EV-C109							2	5	7
EV-C117						1	0		1
PV-2			1		1				2
PV-3				1	1				2
EV-D68		4	25		3	1	23	1	57
Total no.	44	33	72	43	42	36	84	11	365

### EV genotypes in vesicular fluid.

The only genotypes found in vesicular fluids were coxsackievirus CV-A6 and EV-A71. All five patients were children with HFMD.

### EV genotypes in blood samples.

Seven of eight EVs detected in plasma were also detected in another clinical material, i.e., the same EV type was detected in CSF once, in feces three times, and in respiratory materials four times.

The eight patients with positive plasma samples presented with neonatal sepsis three times (echovirus 9 [E-9] twice and CV-B1 once), neonatal hepatitis once (E-19), neonatal myocarditis twice (CV-B1 and CV-B3), HFMD once (CV-A6), and one pregnant woman presented with gastrointestinal symptoms and fever (CV-A9).

### EV genotypes in CSF.

**FIG 2:**
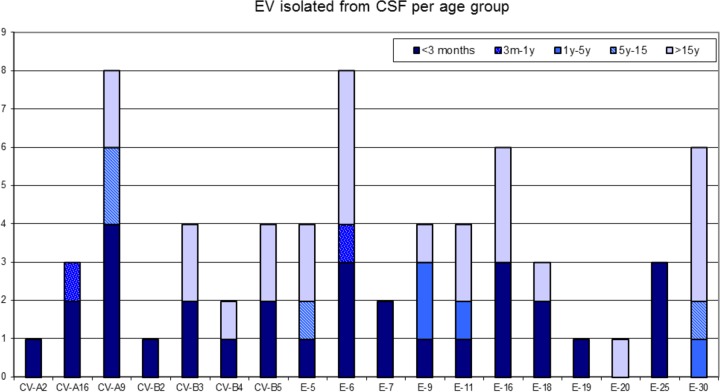
Age distribution of the enterovirus genotypes detected in cerebrospinal fluid. E, echovirus; EV, enterovirus; CV, coxsackievirus.

CV-A9 and E-6 were each detected in CSF eight times during this study. CV-A9 however was more prevalent in materials other than CSF during the study period, with 21 positive tests from 16 disease episodes, compared to E-6, which was detected 15 times from 12 disease episodes (Fig. 2).

EV-A was only detected in 5 out of 65 CSF samples (7.7%). All but one patient were neonates with sepsis and symptoms of meningitis. The one older infant presented with symptoms of viral meningitis. Neurological infections in older children (>1 year) and adults were caused by EV-B (*P* < 0.002). Viral meningitis with detectable EV in CSF was seen in 25 adults (>15 years old) and 40 children. The median age of the adults was 35 (range, 21 to 62).

### EV genotypes in feces.

The most detected genotypes in feces were CV-A6 (*n* = 17), CV-B3 (*n* = 15), CV-A16 (*n* = 14), and E-25 (*n* = 14) (Fig. 1). Although enterovirus may have contributed to gastrointestinal symptoms in these mostly immunocompromised patients, there were no clearly identifiable cases of enterovirus gastrointestinal disease.

EVs from fecal samples were also detected in at least one other clinical material in 21 cases; EVs detected in fecal samples made up at least half of the total number of isolates for most genotypes, excluding echovirus E-5, E-6, E-9, E-18, E-20, and E-30, of which the majority of positive tests were in CSF. In addition, CV-B4, CV-A21, EV-C109, and EV-D68 were predominantly detected in respiratory samples.

### Detection of poliovirus genotypes.

Four poliovirus isolates were genotyped as poliovirus 2 and poliovirus 3 and were identical to the vaccine strains. These were found in fecal samples of young children who had received an oral polio vaccination. All four children were referred to our hospital with chronic bowel problems. None were immunocompromised.

### EV genotypes in respiratory materials.

EVs belonging to all four species A to D were detected in respiratory materials. Seven percent (*n* = 7) of EVs in respiratory materials belonged to species A, 23% (*n* = 22) belonged to species B, 13% (*n* = 12) belonged to species C, and 57% (*n* = 55) belonged to species D. The most prevalent EV type in respiratory materials during our study period was EV-D68 (*n* = 57 isolates from 55 disease episodes). Two periods were observed during which many patients presented with respiratory disease caused by EV-D68. These upsurges in 2010 and 2014 were described separately (22, 29). Of note, in spite of EV-D68 being the EV with the highest circulation in our study population during the observed period, this virus was detected in only 3 fecal samples. All three patients had symptoms of respiratory illness beside gastrointestinal symptoms, which prompted the testing of a fecal sample.

**FIG 3:**
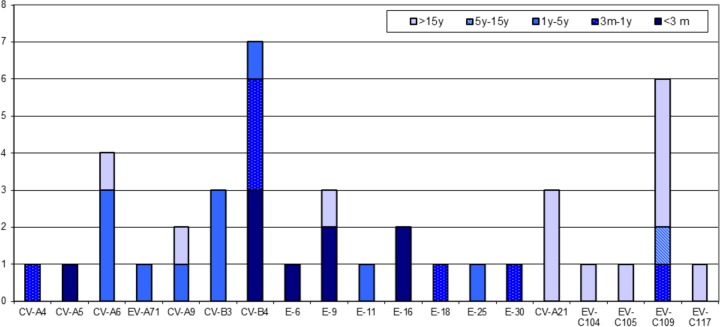
Age distribution of the enterovirus genotypes detected in respiratory materials. E, echovirus; EV, enterovirus; CV, coxsackievirus.

The second most frequently detected EV type in respiratory materials was CV-B4 (7 times) (7%). All patients with this virus were small children between the ages of 2 weeks and 4 years (Fig. 3).

Species C EVs (excluding the four polio vaccine strains) were predominantly detected in respiratory samples. Although the overall prevalence of viruses belonging to this species was very low during this study, i.e., 19 positive tests, 12 (63%) of these were in respiratory samples. Also, while respiratory infections associated with EV-A, EV-B, and EV-D species viruses mainly affected children (<5 years old), respiratory infections associated with EV-C species viruses mainly affected adults (*P* < 0.002).

### Clinical characteristics of patients with C-group enteroviruses.

In view of the previously reported near absence of species C EVs in the Netherlands (30), finding 19 non-polio EV-C genotypes was notable. Moreover, during the study period, a total of 9 EVs were detected that are still considered to be rare. EV-C104, EV-C105, and EV-C117 were each detected once, and EV-C109 was detected 7 times from 6 patients. The patients with EV-C105 and EV-C117 were previously healthy individuals, and the patient with EV-C104 had a hematological malignancy. The six patients with EV-C109, four adults and two children, all presented during the winter of 2014 to 2015. Four out of six had coinfections, and five out of six had underlying diseases (Table 2). One patient had clinical signs and symptoms of viral meningitis with headache, nausea, and vomiting. A CSF investigation showed an elevated leukocyte count (32 × 10^6^/liter), and enterovirus was detected in CSF, although the amount of enterovirus RNA in the CSF was insufficient for sequencing. EV-C109 was subsequently detected in a respiratory specimen of this patient.

**TABLE 2:** Clinical characteristics of the patients who presented with infections associated with EV-C

EV type	Sex^*a*^	Age	Date	Material	Clinical presentation	Coinfections	Underlying condition
EV-C109	M	7 yr	13 December 2014	Respiratory	Mild respiratory illness and vesicles	CV-A6	Renal transplantation
EV-C109	F	35 yr	26 December 2014	Respiratory	Fever, cough	Influenza A	Cyclical neutropenia
EV-C109	F	6 mo	28 January 2014	Respiratory	Fever/respiratory distress	HMPV	None
EV-C109	M	32 yr	1 February 2015	Respiratory	Viral meningitis^*b*^	None	Lymphoma
EV-C109	F	59 yr	3 February 2015	Respiratory/feces	Nausea, vomiting	Norovirus	Lung transplantation
EV-C109	F	62 yr	17 February 2015	Respiratory	Mild respiratory illness	None	Lung transplantation
CV-A21	F	17 yr	8 August 2013	Feces	Diarrhea	Adenovirus	Liver transplantation
CV-A21	M	59 yr	19 September 2013	Respiratory	Cold + reduced lung function	None	Lung transplantation
CV-A21	F	38 yr	5 November 2013	Respiratory	Asthma exacerbation	None	Asthma
CV-A21	M	52 yr	10 December 2013	Respiratory	SOB^*c*^, pleuritic pain, pericarditis	None	Lymphoma
EV-C117	M	27 yr	23 December 2013	Respiratory	Mild cold	None	None
EV-C104	F	10 yr	2 January 2014	Respiratory	Seizure	None	Leukemia
EV-C105	M	19 yr	3 September 2014	Respiratory	Pneumonia	Streptococcus pneumoniae	None

a F, female; M, male.

b This patient, undergoing treatment for malignant lymphoma, presented with symptoms of viral meningitis and elevated lymphocyte count in cerebrospinal fluid. Enterovirus was detected in cerebrospinal fluid but with an insufficient load for genotyping. EV-C109 was detected in a sample of respiratory material.

c SOB, shortness of breath.

Three adult patients had respiratory infections with CV-A21. All three had underlying medical conditions, and all three presented in the same period in 2013 (Table 2). CV-A21 was detected in a fecal sample from one additional patient, a liver transplantation patient who had a coinfection with adenovirus. CV-A1, CV-A11, CV-A20, and CV-A22 were only detected in feces and were presumably incidental findings. In our multiplex screening assay, only the EV-C117 was detected by the rhinovirus test as well as the EV test.

### Phylogenetic analysis of species C enteroviruses.

**FIG 4:**
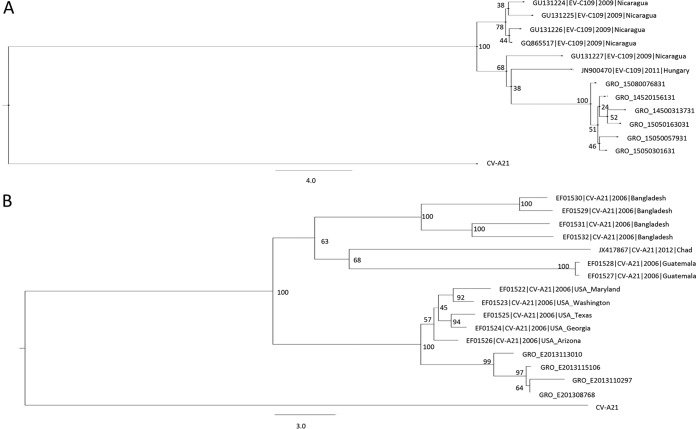
Neighbor-joining phylogeny showing the relationships among the 6 EV-C109 strains from this study (A) as well as the 6 strains that are available in GenBank, based on the alignment of and partial VP1 (322 to 325 nucleotides [nt]) sequences. (B) The neighbor-joining phylogenetic relationships among the 4 CV-A21 strains from this study and reference strains from GenBank. Trees were constructed using BioNumerics software. Bootstrap values (percentage of 1,000 pseudoreplicate data sets) are shown at the nodes. Bars represent the genetic distance. A strain name indicates a GenBank accession number/country or area/year of isolation. GRO, these sequences have been identified as part of this present study. Bar, nucleotide distance as substitutions per site.

The VP1 sequences of the six EV-C109 detections were obtained to determine the homology with the EV-C109 strains available in GenBank by the neighbor-joining method using BioNumerics software. The VP1 dendrogram (Fig. 4A) shows that the six EV-C109 detections from this study are clustered together (strains identified by GRO prefix in all phylogenetic trees) with a nucleotide divergence of less than 8%, with the nearest previously described strain from Hungary (23). Among the local strains from this study, the nucleotide divergence is less than 2.5%.

By comparison, the VP1 sequences of the four local CV-A21 detections (also identified by the GRO prefix) were obtained to determine the homology among these strains and strains available in GenBank. Nucleotide divergence within the local CV-A21 cluster is 0.5% to 5%, whereas nucleotide divergence between the local CV-A21 strains and the closest previously published strains is less than 7%. The dendrogram (Fig. 4B) shows that the CV-A21 strains are most closely related to strains isolated in 2006 in the United States (3).

**FIG 5:**
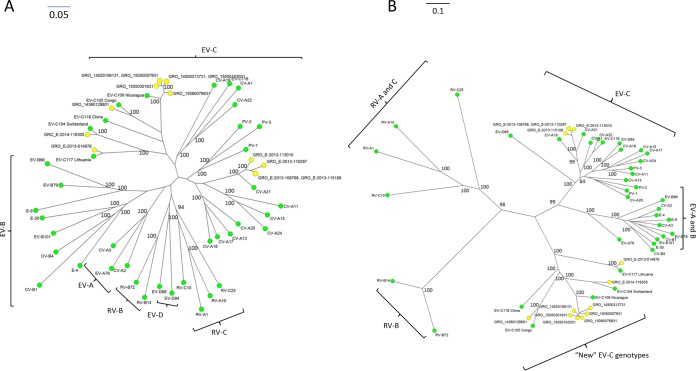
Phylogenetic relationships based on partial VP1 sequences (A) and 5′-UTR sequences (B) of the EV-C isolates from this study, identified by GRO (yellow dots), compared with prototype strains of EV-A, EV-B, EV-C, EV-D, and rhinovirus (RV)-A, RV-B, and RV-C (green dots). Relationships were constructed using the neighbor-joining algorithm. Genotype and country of isolation of each reference strain are indicated. Bootstrap values (percentage of 1,000 pseudoreplicate data sets) are shown at the nodes. Bar, nucleotide distance as substitutions per site.

The VP1 sequences of the single detections of EV-C104, EV-C105, and EV-C117 were obtained, and all of these were used for the construction of the VP1 unrooted phylogenetic tree in Fig. 5A. The VP1 neighbor-joining tree shows the grouping of all species C viruses from the present study, clustering with their prototypic counterparts, within the clade of EV-C.

Because significant divergence has been shown in the 5′ UTR of the new EV-C viruses, EV-C104, EV-C105, EV-C109, and EV-C117, compared to the “classical” EV-C viruses, we also sequenced this region in all of the EVs included in this study. The unrooted neighbor-joining tree shows that these new EV-C viruses form a separate clade, while the four CV-A21 isolates cluster with the other “classical” EVs of species C (Fig. 5B).

## DISCUSSION

With this report, we present the data from the routine genotyping of all EVs detected in our tertiary care hospital between January 2008 and April 2015. Forty different EV genotypes were detected in our clinical virology laboratory. During the study period, EV diversity was dominated by two upsurges of EV-D68 in respiratory samples, one in 2010 and one in 2014, which together with nine sporadic cases in the remaining study years account for 16% of the typeable isolates. Interestingly, only three fecal samples during the study period had detectable EV-D68. All three patients also had respiratory illness, most likely caused by this virus. While in this study most EVs detected in fecal samples represented background circulation, it is interesting that this virus was not detected as an incidental finding in patients presenting with other symptoms. Previous studies that have investiged EV-D68 report that the detection of EV-D68 appears to be restricted to respiratory materials (30). However, no EV genotype prevalence studies exist that include years with EV-D68 outbreaks. Considering the very low overall prevalence of EVs in respiratory materials (0.7%), EVs did not appear to be important respiratory pathogens outside of the two EV-D68 outbreak years. Moreover, all EVs probably replicate to some extent in the nasopharyngeal epithelium and may be detectable in respiratory samples (1). Nevertheless, the genotypes that we detected in respiratory materials were significant. While 41 respiratory materials were positive for EVs other than EV-D68, 12 (29%) of these were EV-C genotypes. Twenty-three species C EVs were detected, which is remarkable because similar studies to ours in the past have found very few, if any, EVs belonging to the EV-C group (31, 32). Many of these studies, however, did not include respiratory materials, which is where most species C EVs were found in this study. The classical CV-A21 was detected in respiratory secretions of three patients who all presented with severe respiratory illness in the fall of 2013. One instance of CV-A21 was detected in the feces of a patient with diarrhea, probably an incidental finding. CV-A21 is currently a well-recognized cause of respiratory problems (33). The sequence analyses of the CV-A21 isolates in this study show that they are closely related and that they are very similar to CV-A21 circulating in North America (3).

Additionally, the presence of the new EV-C genotypes EV-104, EV-105, EV-109, and EV-117 is of importance. To date, very few viruses of these genotypes have been reported worldwide. Of EV-C104, EV-C105, EV-C109, and EV-C117, the total number of isolates described worldwide is less than 10 each (19–25). Especially for EV-C109, which appears to be extremely rare, it is remarkable that six patients with this virus were found in a relatively small study. It is possible that EV-C109 was only recently introduced in Europe or that we experienced a small upsurge of an otherwise low pathogenic virus, which was only found because of the vulnerable patient population in our hospital. Yet, two other reasons have to be considered.

This study shows that the 5′ UTR, which is frequently used as a target for molecular tests, is significantly different for these viruses, confirming what has been reported by other researchers (12, 16, 17). The divergence in the 5′ UTR is likely to lead to difficulties in detecting these viruses. Undeniably, this has already been an issue in one of the AFP cases (20). Another reason may be the exclusion of respiratory materials in many epidemiological studies such as ours (31, 32).

Because of the particular patient population in our hospital and the relatively small sample size, it is not possible to draw any conclusions about the importance of respiratory EVs other than EV-D68 at this time. The exceptional patient population of this study is exemplified by the low number of vesicular fluids included in this report, as HFMD is typically a diagnosis made in general practice and also by the clinical characteristics of the patients presenting with respiratory infections associated with EV-C. The data generated in this study are therefore not suitable for determining the prevalence of viruses.

A representative picture of genotype prevalence and associated disease can only be obtained if different health care providers collaborate in collecting samples and data. Therefore, we have started a regional surveillance network that is aimed at genotypic analysis of all EVs detected in the northern part of the Netherlands. Ideally, this regional network would be linked to other regional networks, spanning a country of even multiple countries (34). Recently, a network such as this that is aimed at rapid detection and genotyping of EV-D68 was formed throughout Europe, showing the preparedness and the motivation of many laboratories and health institutions to participate in a Europe-wide network (11). Good detection assays for EVs are an obvious prerequisite for a functioning network.

In conclusion, this study shows the circulating EV genotypes in a tertiary care hospital in the Netherlands. The detection of a large number of EVs in respiratory materials in patients with respiratory illnesses confirms that some EVs are considerable respiratory pathogens. Moreover, although they are not associated with a major impact on public health, our study shows that newly described EVs were found to circulate in the Netherlands when respiratory samples were tested.
